# Spontaneous ventilation anesthesia combined with uniportal and tubeless thoracoscopic sympathectomy in selected patients with primary palmar hyperhidrosis

**DOI:** 10.1186/s13019-022-01917-4

**Published:** 2022-07-15

**Authors:** Guang-Qiang Shao, Da-Zhi Pang, Ji-Tian Zhang, Hong-Xia Wang, Tai-Yang Liuru, Zhi-Hai Liu, Ya-Nan Liang, Jing-Si Liu

**Affiliations:** 1 Division of Thoracic Surgery, Department of Surgery, The University of Hong Kong-Shenzhen Hospital, 1 Haiyuan 1st Road, Futian District, Shenzhen City, Guangdong Province China; 2 Division of Respiratory and Critical Care Medicine, The University of Hong Kong-Shenzhen Hospital, Shenzhen, China; 3 Division of Pediatric Surgery, Department of Surgery, The University of Hong Kong-Shenzhen Hospital, Shenzhen, China

**Keywords:** Nonintubated VATS, Single 5 mm port, Sympathectomy, Spontaneous ventilation anesthesia (SVA), Tubeless, Primary palmar hyperhidrosis (PPH)

## Abstract

**Background:**

To assess the feasibility and safety of tubeless video-assisted thoracoscopic sympathectomy (VATS) with a single 5 mm port under nonintubated, intravenous anesthesia with spontaneous ventilation in selected patients with primary palmar hyperhidrosis (PPH).

**Methods:**

Adults (aged between 18 and 60 years) with moderate or severe PPH symptoms were enrolled. Demographic information and clinical data were obtained from 172 consecutive patients undergoing thoracoscopic surgery for PPH from March 2014 to December 2020. The primary outcomes were the rate of complications, including death, and the intraoperative conversion rate to 3-port VATS. The secondary outcomes were the conversion rate to intubated anesthesia during the operation and the surgical duration and pain score of postoperative day 0.

**Results:**

In total, 172 patients were included with 88 males and 84 females. The median age was was 25 years (IQR:21–30 years). No mortalities or major morbidities occurred in any patient. The overall median surgical duration was 53 min (IQR:37–72 min). The median length of postoperative hospital stay was one day (IQR:one–one day). The median pain score of POD0 was 2 (IQR:2–2). Intraoperative conversion to 3-port VATS followed by drainage tube insertion occurred in one (0.6%) patient due to extensive pleural adhesions. No patients required conversion to intubated anesthesia during surgery. No postoperative mechanical ventilation was noted in any patient.

**Conclusions:**

For selected patients with PPH, tubeless VATS with a single 5 mm port using spontaneous ventilation anesthesia can be considered a feasible and safe operation. The surgical wound is extremely small and the operation time is shorter than the conventional technique.

*Trial registration* This study was in conformity with the Declaration of Helsinki, and was approved by the National Ethics Committee of the University of the Hong Kong-Shenzhen Hospital (Approval number: [2020]70). We registered the study in the Chinese Clinical Trial Registry (Registration number: ChiCTR2100049063) in 2021.Informed consent was collected from all the participants of this study. URL for this clinical trial registration is: https://www.chictr.org.cn/index.aspx.

## Background

Primary palmar hyperhidrosis (PPH) is a disorder of unknown etiology characterized by excessive sweating of the hands, feet, and armpits. This disorder can have serious social, emotional, and professional impacts, especially in young adult and adolescent patients, for whom early detection and management can significantly improve quality of life [1].

In the past, conventional treatments such as oral antiperspirants or sprays were common, but they were later suggested to be unsatisfactory. In recent years, video-assisted thoracoscopic sympathectomy (VATS) has been widely used due to its low morbidity, long-term satisfactory results, and ability to significantly improve quality of life [2–8].

Moreover, spontaneous ventilation anesthesia (SVA) and uniportal and tubeless approaches represent remarkable developments in VATS. The advantages of these techniques include a smaller incision, less postoperative pain and wound paresthesia, avoidance of intubation-related airway trauma and postoperative cough, shorter hospital stay, less damage to lung function, and better experience and acceptability [9–11]. However, a combination of all these techniques for VATS has not yet been reported.

This study aimed to assess the feasibility and safety of tubeless VATS with a single 5 mm port under nonintubated, intravenous anesthesia with spontaneous ventilation without the placement of a chest tube postoperatively in selected patients with PPH.

## Methods

This study was approved by the Institutional Ethical Review Board of the University of Hong Kong-Shenzhen Hospital, Guangdong Province, China. This was a retrospective review of surgical data obtained from March 2014 to December 2020. The inclusion criteria were as follows: age ≥ 18 years and ≤ 60 years, moderate or severe symptoms of PPH, body mass index < 28, and resting heart rate > 55 beats/min. None of the patients were treated with any medications for hyperhidrosis during this study. Patients with cardiac disease, cardiac arrhythmia, pacemakers, or cerebral contusion were excluded. Informed consent was obtained from all the patients before the VATS procedure. The surgery was performed by surgeons from the thoracic department with the help of experienced anesthesiologists using intubated or nonintubated, intravenous anesthesia with spontaneous ventilation.

### Statistical analysis

SPSS for Windows version 25.0 (SPSS Inc., Chicago, Illinois, USA) was used for statistical analysis of the data obtained in this study. The data were described as the mean (standard deviation), median (25% quantile, 75% quantile), or numbers (percentages) according to the variable type and distribution. The normality of the distribution was confirmed with the Kolmogorov–Smirnov test. For the data of this study do not follow to the normal distribution, they were presented as median (25% quantile and 75% quantile), or numbers (percentages). Chi-squared tests, Fisher’s exact tests, or Kruskal–Wallis tests were used for group comparisons. In order to further study the relationship between type of anesthesia, number of surgical port and the surgical duration, two models had been built as follow, model 1 adjusted age, sex, smoking history and number of surgical port, model 2 adjusted age, sex, smoking history and type of anesthesia. A two-side P value of < 0.05 was considered statistically significant.

### Anesthesia technique

In the operating room, the patients’ heart rate, electrocardiogram, blood pressure, blood oxygen saturation, bispectral index (BIS, Medtronic, Minneapolis, MN, USA), and palm temperature were continuously monitored by the anesthesiologists.

**Fig. 1:**
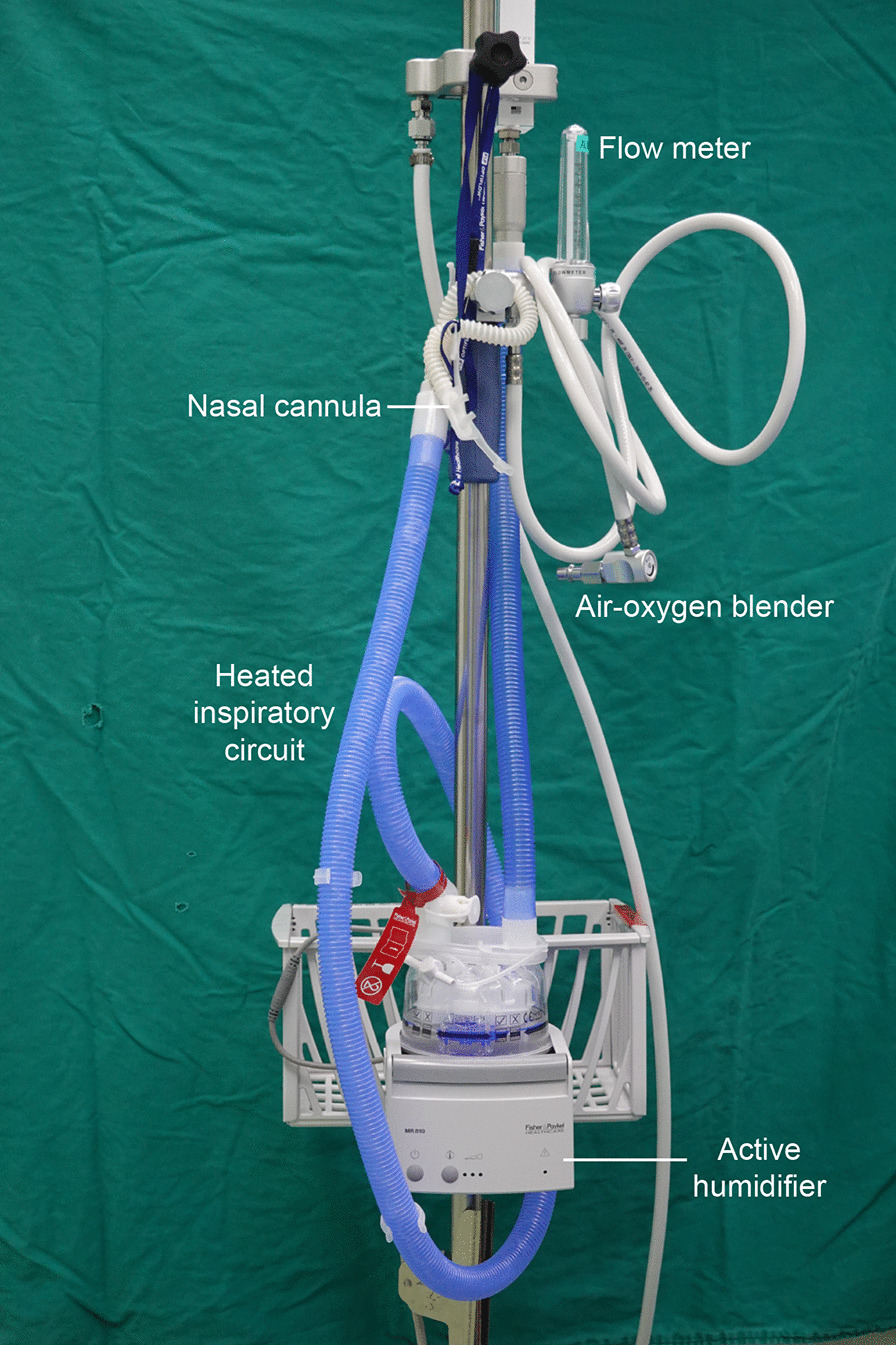
The HFNC system includes a flow meter, an air-oxygen blender, a humidifier, a heated circuit, and nasal prongs

**Fig. 2:**
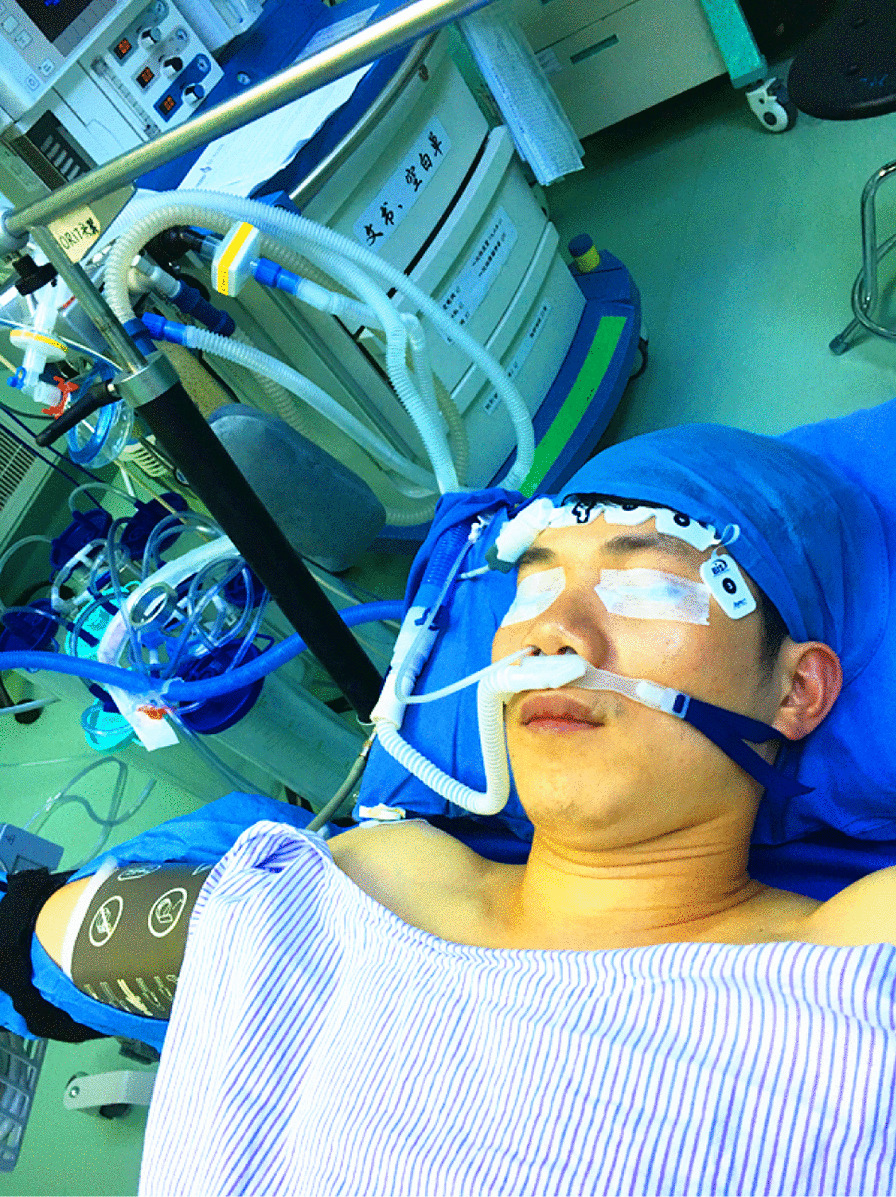
A male patient was placed in a semi-Fowler's position and the HFNC system was connected with his nasal opening

After the establishment of intravenous infusion, propofol was administered in combination with sufentanil. a double-lumen endotracheal intubation technique or non-intubation technique (spontaneous ventilation anesthesia) was performed according to the experience of different anesthesiologists. A high-flow nasal cannula (HFNC) system was used to accomplish spontaneous ventilation anesthesia in our center which included a flow meter, an air-oxygen blender, a heated circuit, a humidifier, and nasal prongs (Fig. 1) was placed at the patient’s nasal opening (Fig. 2).

Spontaneous breathing was maintained at a respiratory rate of 12–20 per minute, and the BIS was used to monitor the depth of sedation, which ranged from 40 to 60. If any complications (e.g., bleeding, hypoxemia, or hypercapnia) occurred during the operation and could not be resolved as soon as possible, double-lumen endotracheal intubation technique was used to achieve lung isolation without any hesitation.

### Surgical technique

**Fig. 3:**
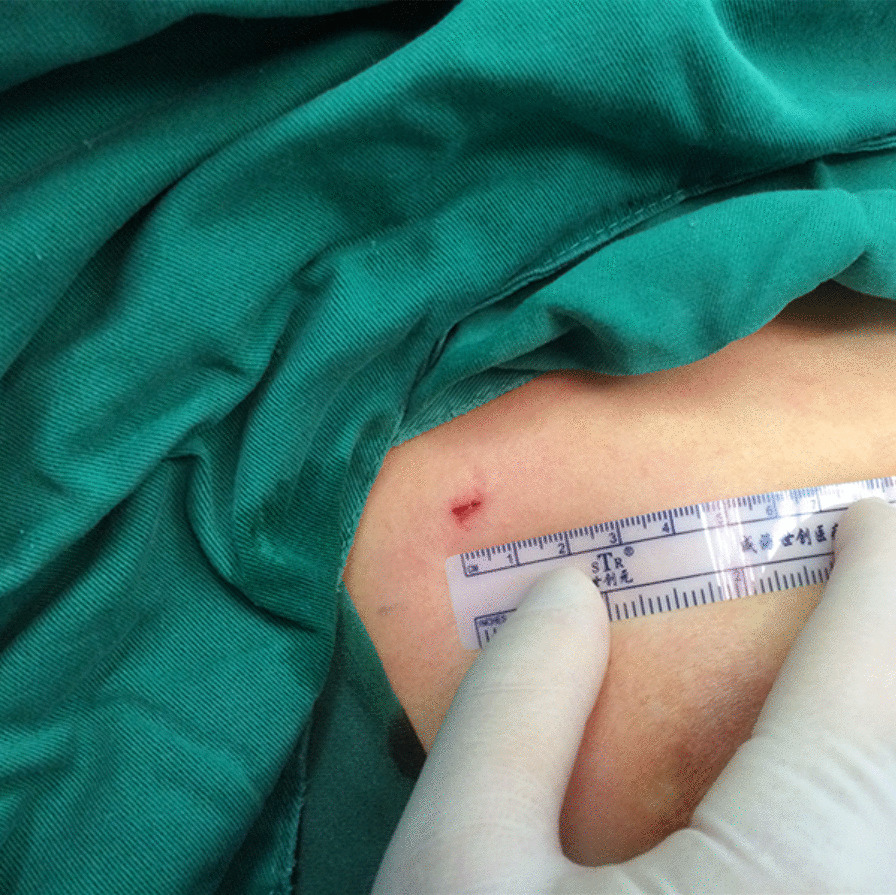
A 5-mm incision was made at the 3rd intercostal space between the anterior axillary line and midaxillary line

**Fig. 4:**
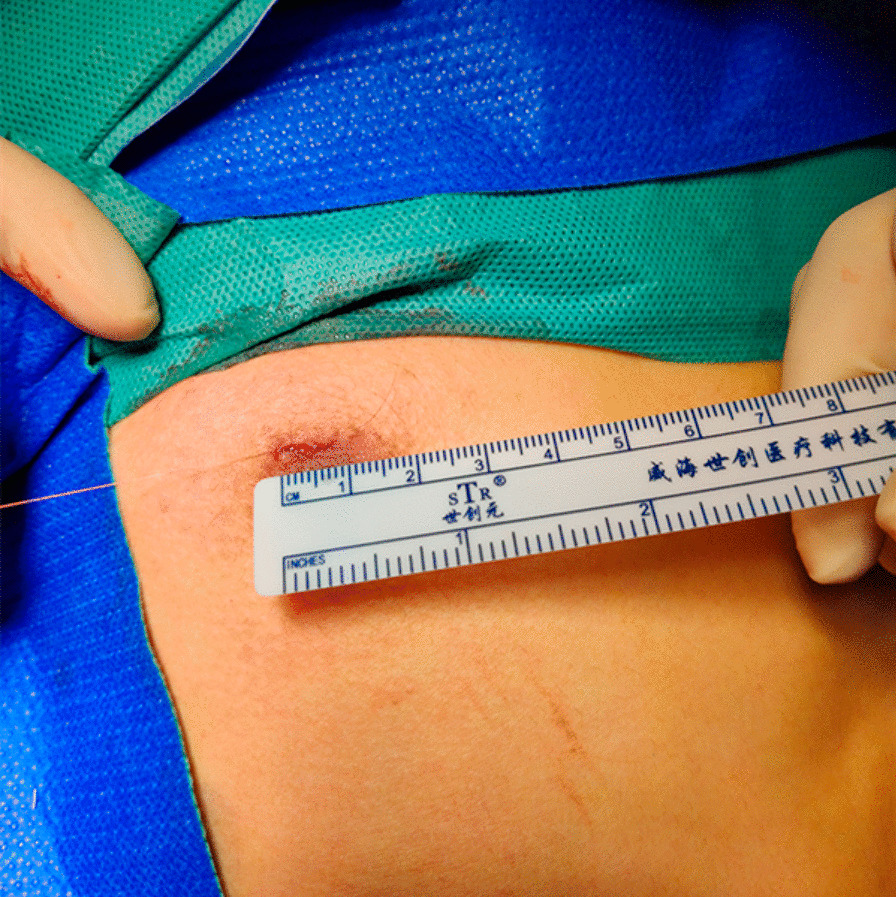
A 5-mm incision was made on the right side at the periareolar region

All patients were placed in a semi-Fowler's position with both arms abducted at 90°. The chest was prepped and draped in a sterile fashion. Starting from the right side, 5 mL of local anesthesia (2% lidocaine) was subcutaneously injected to minimize pain. Then, according to the experience of different surgeons, the conventional three-port VATS approach is as follows, a 3-mm incision was made as the camera port in the mid-axillary line of the 4th intercostal space, and another two 3-mm incision was made as the operating ports in the mid-axillary line of the 2th intercostal space and the anterior axillary line of the 4th intercostal space. Alternatively, single-port VATS approach is as follows, a single 5-mm incision was made at the 2nd or 3rd intercostal space between the anterior axillary line and midaxillary line under the armpit (Fig. 3) or the periareolar region (male patients only) (Fig. 4). A 3-mm thoracoport was inserted into the pleural space, and a 30° thoracoscopy (3 mm) lens was placed for confirmation. Then, the side hole on the 3-mm thoracoport was connected to the carbon dioxide pump, and carbon dioxide gas was continuously pumped into the chest cavity, collapsing the lung to gain better exposure. During this procedure, the flow rate was maintained at 1–2 L/min, and the pressure was 5 mmHg. If pleural adhesions were found, an electrocoagulation hook was used to release the adhesions. Once the location of the R3 or R4 sympathetic nerve chain on the surface of the 3rd or 4th ribs was identified, respectively, a hook was used to cut the R3 or R4 sympathetic nerve chain horizontally. To eliminate possible exposure of the Kuntz bundle, a cautery was used inwards and at least 2 cm outwards. If the palmar skin became dry and the temperature increased by more than 1 °C from baseline, the treatment was considered to be effective. After hemostasis was confirmed, an 8F drainage tube was inserted into the chest cavity, and the subcutaneous tissue was subsequently closed. Negative pressure suction and the Valsalva maneuver (the patient was ventilated at 20 cm H2O of maximum inspiratory pressure) were implemented to promote lung re-expansion. The drainage tube was removed rapidly once the water seal test confirmed the absence of an air leakage. Finally, the skin was closed with a 5–0 Vicryl suture. The same procedure was repeated on the left side.

### Postoperative management

The patients were transferred back to the general ward following a short recovery period in the resuscitation room. After four hours of close monitoring, a chest X-ray was obtained, and the patients were discharged if no remarkable findings were observed.

### Data collection and follow-up

The demographic data of the patients, surgical duration, blood loss, length of hospital stay, resolution of palmar hyperhidrosis, and complications were noted. Postoperative complications included pulmonary and pleural complications, such as air leakage, empyema, respiratory failure, bleeding. The follow-up assessments were performed through direct examination by a clinician at the outpatient clinic as well as through questionnaire surveys and telephone calls.

## Results

### Patients’ characteristics

A total of 172 eligible patients, identified by a retrospective chart review, were enrolled in the study, and analysis of the surgical data revealed no mortalities or major morbidities. The sample consisted of 88 males and 84 females, with a median age of 25 years (IQR:21–30 years). 9 patients (5.2%) had a history of smoking. 28 patients (16.3%) underwent endotracheal intubation and 144 patients (83.7%) underwent non-tracheal intubation or spontaneous ventilation anesthesia (SVA). The VATS approach was single-port in 95 cases (55.2%), and three-port in 77 cases (44.8%). The overall median surgical duration was 53 min (IQR:37–72 min). The median length of postoperative hospital stay was one day (IQR:one–one day). The median pain score of POD0 was 2 (IQR:2–2). The demographic and clinical characteristics of patients are shown in Table 1.

**Table 1:** Characteristics of the study population

Total patients (n = 172)	
Age (MD,IQR), years	25 (21,30)
Male (n, %)	88 (51.2%)
Female (n, %)	84 (48.8%)
Smoking history	
Smoker (n, %)	9 (5.2%)
Non-smoking (n, %)	163 (94.8%)
Type of anesthesia	
Intubation (n, %)	28 (16.3%)
Spontaneous ventilation anesthesia (n, %)	144 (83.7%)
Surgical approach	
Single-port VATS (n, %)	95 (55.2%)
Three-port VATS (n, %)	77 (44.8%)
Single-port convered to 3-port VATS (n, %)	1 (0.6%)
Surgical duration (MD, IQR), min	53 (37, 72)
Blood loss during the operation (MD, IQR), ml	2 (1, 2)
Pleural adhesion (n, %)	6 (3.5%)
Mild	3 (1.7%)
Moderate	1 (0.6%)
Severe	2 (1.2%)
Chest dragainge insertion during the surgery (n, %)	3 (1.7%)
Pneumothorax on POD0 Chest X ray (n, %)	
0–10%	155 (90.1%)
11–20%	10 (5.8%)
21–30%	5 (2.9%)
31–40%	2 (1.2%)
Chest dragainge insertion after the surgery (n, %)	1 (0.6%)
Length of postoperative hospital stay day (MD, IQR)	1 (1,1)
Less than 24 h	153 (89.0%)
More than 24 h	19 (11.0%)

Data are expressed as median (25th, 75th percentiles) or n (%)

### Primary outcomes

No mortality or major morbidity occurred in any patient. 6 patients (3.5%) experienced complications, included 4 cases of air leaks, 2 cases of intraoperative bleeding. Of the 3 patients with air leaks, moderate to severe pleural adhesions were found during the operation. All of them underwent three-port VATS approach in the SVA technique group followed by drainage tube insertion, including a temporary change to three-port VATS approach in one patient scheduled for single-port VATS approach. One patient in the SVA technique group using single-port VATS approach needed a postoperative indwelling chest tube because of postoperative delayed pneumothorax. 2 patients with intraoperative bleeding in the SVA technique group using three-port VATS approach were all venous hemorrhage after inadvertently injury to venules, and there was no rebleeding after electrocoagulation for hemostasis.

### Secondary outcomes

No patients required conversion to intubated anesthesia during surgery. No postoperative mechanical ventilation was noted in any patient. The overall median surgical duration was 53 min (IQR:37–72 min). In all population, the patients in intubated group had a longer length of surgical duration than those in SVA group. Furthermore, in SVA group those patients who accepted single-port VATS approach present a shorter surgical duration compared with those accept the conventional three-port VATS approach (45 min vs. 56 min, P < 0.001, detail are shown in Table 2). After we adjusted age, sex, smoking history and number of surgical port, the surgical duration was significantly shorter in SVA group (ajusted odds ratio 0.23, 95% confidence interval 0.07–0.68, P = 0.008). Similarly, after we adjusted age, sex, smoking history and type of anesthesia, the surgical duration was significantly longer in the conventional three-port VATS approach group compared with single-port VATS approach group (ajusted odds ratio 2.8, 95% confidence interval 1.5–5.3, P = 0.001). The details are shown in the Table 3.

**Table 2:** Comparison of outcomes between patients in different disease subgroups

Outcomes	Total	Intubation N = 28			SVA N = 144			P value
		Single-port VATS N = 3	Three-port VATS N = 25	P value	Single-port VATS N = 92	Three-port VATS N = 52	P value	
Complications (n, %)	6 (3.5%)	0	0	–	1 (1.09%)	5 (9.6%)	0.01	0.05
Surgical duration (MD, IQR), min	53 (37, 72)	72 (58, 74)	68 (56, 77)	0.97	45 (23, 60.5)	56 (45, 83)	< 0.001	< 0.01
Length of postoperative hospital stay day,(MD, IQR)	1 (1, 1)	1 (1, 2)	1 (1, 1)	0.09	1 (1, 1)	1 (1, 1)	0.48	0.07
Pain score of POD0 (MD, IQR)	2 (2, 2)	2 (2, 2)	2 (2, 3)	0.29	2 (2, 2)	2 (2, 2)	0.94	0.06

**Table 3:** Association between variables and surgical duration

Surgical duration	Variable	Odds ratio	95% CI	P-value
Crude	Intubation	Ref	0.06–0.45	< 0.001
	SVA	0.16		
Adjusted model 1	Intubation	Ref	0.07–0.68	0.008
	SVA	0.23		
Crude	Single-port VATS	Ref	1.5–5.3	0.001
	Three-port VATS	2.8		
Adjusted model 2	Single-port VATS	Ref	1.02–3.98	0.04
	Three-port VATS	2.02		

The association between variables and surgical duration were analyzed using logistic regression models

Adjusted model 1: adjusted age, sex, smoking history and number of surgical port

Adjusted model 2: adjusted age, sex, smoking history and type of anesthesia

## Discussion

VATS is the most effective and long-lasting method to treat palmar hyperhidrosis among the techniques available thus far. With the development of VATS techniques and technology for anesthesia control, VATS is becoming increasingly minimally invasive. In this study, a tubeless approach (without tracheal intubation, urinary catheterization, or postoperative chest drain insertion) was performed in select patients with PPH who underwent VATS. According to our findings, no mortalities or major morbidities occurred in any the study participants.

Most of the patients in our study were young adults and had a desire for improved quality of life [1]. Smaller and more inconspicuous incisions could meet their requirements and increase their satisfaction with the surgery. In recent years, the number of incisions has been reduced from three to one under the armpit or in the periareolar area (male patients only). With the application of thoracoscopy with a 3-mm needle, the incision has become even smaller. Villamizar et al. [9] performed sympathectomy through the axilla this approach and achieved satisfactory results.

**Fig. 5:**
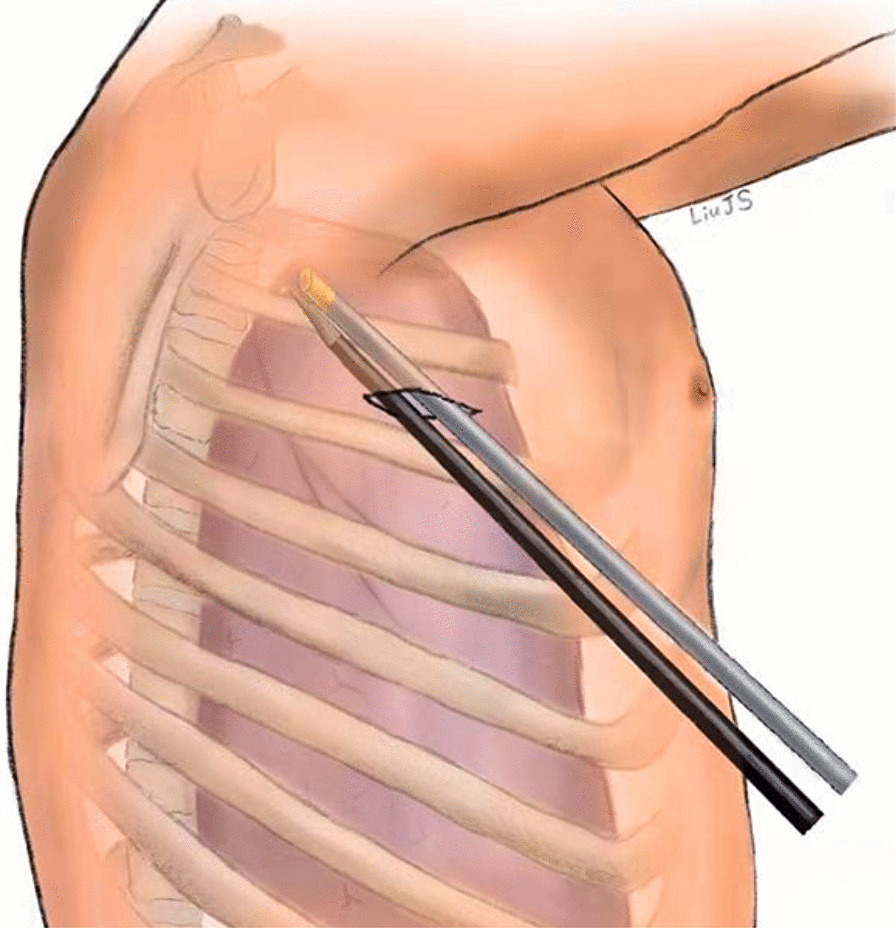
The chief surgeon holds the 3-mm lens and coagulation hook with both hands close together

**Fig. 6:**
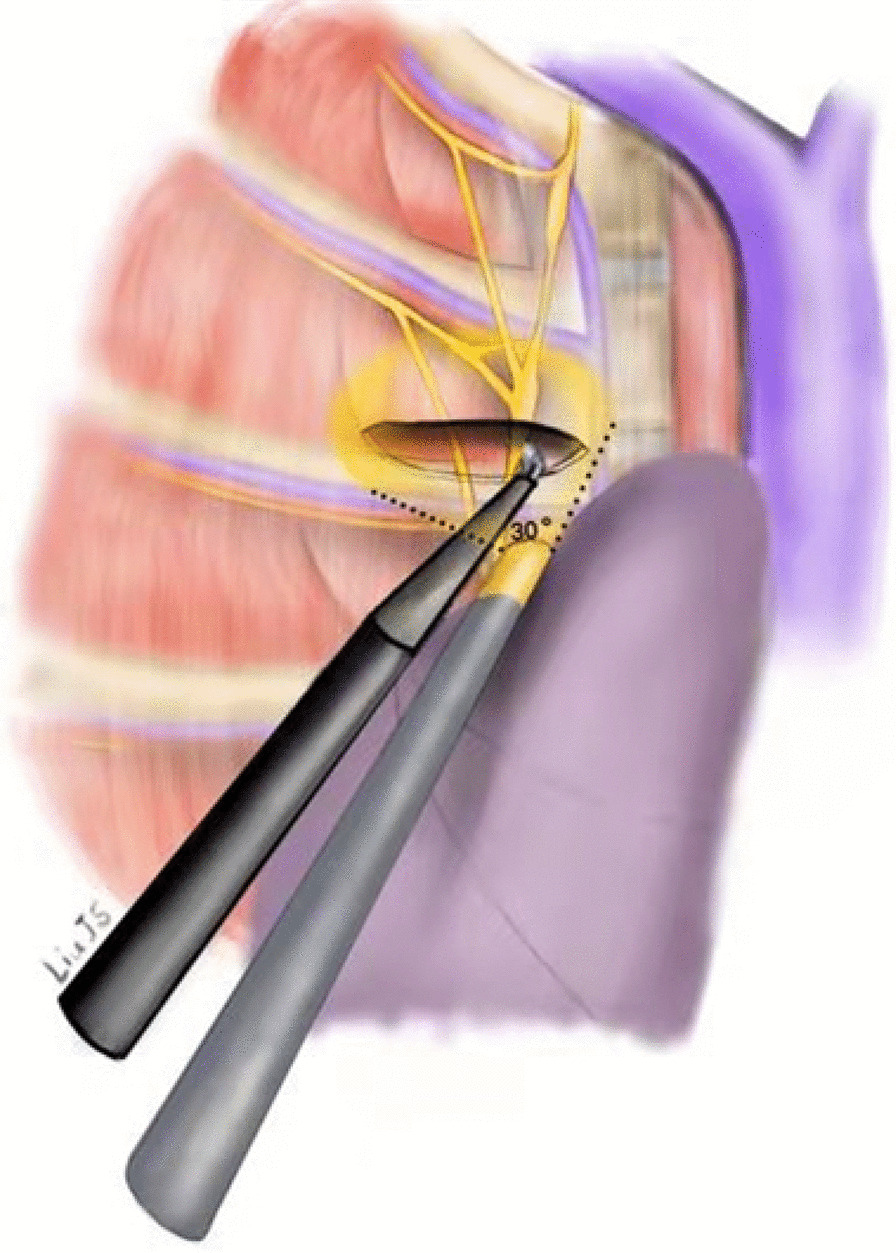
During the operation, we used a 30-degree lens to obtain a better view, reducing the chance that the devices would interfere with one another

However, advantages and disadvantages still exist, and a single 5-mm incision undoubtedly increases the difficulty of the operation. To solve this problem and help improve our surgical skills using just a 5-mm incision, the chief surgeon can hold the 3-mm lens and coagulation hook closely together with his or her hands (Fig. 5) to reduce the chance of the devices interfering with each other and finally use the 30-degree lens to obtain a better view and safely cut the R3 or R4 sympathetic nerve chain (Fig. 6).

In our study, we found that the patients in SVA group those patients who accepted single-port VATS approach present a shorter surgical duration compared with those accept the conventional three-port VATS approach. The reason behind this was that single-port VATS approach can simplify the operation process. In addition, practice can increase the skill level, and experienced hands can perform the procedure better and smoother.

Nowdays, nonintubated anesthesia with spontaneous ventilation has been widely used in VATS. Oxygen can be delivered through a laryngeal mask, facial mask, or HFNC. HFNC oxygen systems have been used in response to many situations, such as hypoxemic respiratory failure, exacerbation of chronic obstructive pulmonary disease (COPD), sleep apnea, acute heart failure, and conditions entailing do-not-intubate orders, as well as for post-extubation or pre-intubation oxygenation [12]. The HFNC system included a flow meter, an air-oxygen blender, a humidifier, a heated circuit, and nasal prongs that connected with the patients’ nasal opening [12]. This system allows the anesthesiologist to set an oxygen flow rate of up to 60 L/min and a fraction of inspired oxygen as high as 100% [13]. Our hospital has accumulated much experience in this field, and HFNC oxygen devices are commonly used at our institution. Our study showed that HFNC oxygen devices provide an effective and feasible anesthesia option for tubeless VATS with a single 5-mm port and was suitable for selected patients who had PPH ranging from 18 to 53 years. Compared with intubated anesthesia, this approach could avoid the residual effects of muscle relaxants, lower the incidence of systemic complications, and thus achieve faster recovery of respiratory muscle function [14]. Moreover, from the data we found that the patients in intubated group had a longer length of surgical duration than those in SVA group. The reason for the difference was that we used the carbon dioxide pump to manufacture artificial pneumothorax, which can made the surgical field exposed faster. In addition, intubation anesthesia was used more frequently in the early stage, our surgical speed and proficiency had constantly improving overtime. The most common complications of HFNC oxygen therapy are hypoxemia and carbon dioxide retention, but these were not noted in this study. To sum up all the above points, we found that both spontaneous ventilation anesthesia technique and single-port VATS technique play an important role in shortening the surgical duration when we analyzed all the data using logistic regression models.

Furthermore, perioperative chest tube insertion is associated with more pain and thus prolongs the hospital stay, weakens pulmonary ventilation function [15], and prolongs early ambulation [16]. In our study, 3 patients (1.7%) in the SVA technique group using three-port VATS approach required chest drainage tube insertion during the surgery, and all of them were associated with moderate to severe pleural adhesions found during the surgery. 153 (89.0%) patients discharged from the hospital within 24 h of surgery. One of the reasons behind this is that we aimed to avoid any damage to the lung tissue or blood vessels during the operation. The other reason is that we used an 8F chest drainage tube as a temporary chest drain to assist in lung expansion and provide negative pressure and performed a water seal test to monitor for air leakages after the operation. Once the absence of an air leakage was confirmed, the 8F chest drainage tube was then removed, and the wound was sutured with 5–0 Vicryl immediately. A postoperative chest X-ray was performed four hours after the surgery to confirm lung expansion and identify possible pleural effusion. Among the included patients, only one of them needed postoperative chest tube insertion for the chest X-ray on the POD1 showed that the volume of pneumothorax on the right side increased from 25 to 75%. The chest tube was removed after the chest X-ray showed no air leakage and this patient discharged from hospital on POD3. The reason for the air leakage may be that mild pleural adhesions were found during the surgery, and the lung tissue was inadvertently injured when the adhesions were separated. In addition, 2 patients with intraoperative bleeding in the SVA technique group were operated by the same junior surgeon using three-port VATS approach, and there was no rebleeding after electrocoagulation for hemostasis. From the above data analysis, we believe that whether it is air leakage or intraoperative bleeding, it is related to the pleural effusion and the experience of different surgeons, rather than anesthesia technique or port numbers.

This study has some limitations that should be noted. First, this study was retrospective in nature with a small sample size and it’s a single center-retrospective study. Second, the sample size of endotracheal intubation group was relatively small and it’s not a randomized controlled trial and needs further follow-up research.

## Conclusions

This study concluded that tubeless VATS with a single 5-mm port using spontaneous ventilation anesthesia was a safe and feasible surgical strategy for selected patients with PPH. The surgical wound is extremely small and the operation time is shorter than the conventional technique.

## Data Availability

The dataset used and/or analyzed during the current study is available from the corresponding author on reasonable request.

## Abbreviations

VATS: Video-assisted thoracoscopic surgery
PPH: Primary palmar hyperhidrosis
SVA: Spontaneous ventilation anesthesia
BIS: Bispectral index
HFNC: High-flow nasal cannula
COPD: Chronic obstructive pulmonary disease
POD: Postoperative day

## References

[1] Atkins JL, Butler PE (2002). Hyperhidrosis: a review of current management. Plast Reconstr Surg.

[2] Henteleff HJ, Kalavrouziotis D (2008). Evidence-based review of the surgical management of hyperhidrosis. Thorac Surg Clin.

[3] Cerfolio RJ, De Campos JR, Bryant AS, Connery CP, Miller DL, DeCamp MM (2011). The society of thoracic surgeons expert consensus for the surgical treatment of hyperhidrosis. Ann Thorac Surg.

[4] Stolman LP (1987). Treatment of excess sweating of the palms by iontophoresis. Arch Dermatol.

[5] Weber A, Heger S, Sinkgraven R, Heckmann M, Elsner P, Rzany B (2005). Psychosocial aspects of patients with focal hyperhidrosis. Marked reduction of social phobia, anxiety and depression and increased quality of life after treatment with botulinum toxin A. Br J Dermatol.

[6] Kuo CH, Yen M, Lin PC (2004). Developing an instrument to measure quality of life of patients with hyperhidrosis. J Nurs Res.

[7] de Campos JR, Kauffman P, Werebe Ede C, Andrade Filho LO, Kusniek S, Wolosker N (2003). Quality of life, before and after thoracic sympathectomy: report on 378 operated patients. Ann Thorac Surg.

[8] Kumagai K, Kawase H, Kawanishi M (2005). Health-related quality of life after thoracoscopic sympathectomy for palmar hyperhidrosis. Ann Thorac Surg.

[9] Villamizar NR, Darrabie MD, Burfeind WR, Petersen RP, Onaitis MW, Toloza E (2009). Thoracoscopic lobectomy is associated with lower morbidity compared with thoracotomy. J Thorac Cardiovasc Surg.

[10] Paul S, Altorki NK, Sheng S, Lee PC, Harpole DH, Onaitis MW (2010). Thoracoscopic lobectomy is associated with lower morbidity than open lobectomy: a propensity-matched analysis from the STS database. J Thorac Cardiovasc Surg.

[11] Cao C, Manganas C, Ang SC, Yan TD (2012). A meta-analysis of unmatched and matched patients comparing video-assisted thoracoscopic lobectomy and conventional open lobectomy. Ann Cardiothorac Surg.

[12] Nishimura M (2016). High-flow nasal cannula oxygen therapy in adults: physiological benefits, indication, clinical benefits, and adverse effects. Respir Care.

[13] Kang BJ, Koh Y, Lim CM, Huh JW, Baek S, Han M (2015). Failure of high-flow nasal cannula therapy may delay intubation and increase mortality. Intensive Care Med.

[14] Cui F, Liu J, Li S, Yin W, Xin X, Shao W (2016). Tubeless video-assisted thoracoscopic surgery (VATS) under non-intubated, intravenous anesthesia with spontaneous ventilation and no placement of chest tube postoperatively. J Thorac Dis.

[15] Refai M, Brunelli A, Salati M, Xiumè F, Pompili C, Sabbatini A (2012). The impact of chest tube removal on pain and pulmonary function after pulmonary resection. Eur J Cardiothorac Surg.

[16] Ueda K, Sudoh M, Jinbo M, Li TS, Suga K, Hamano K (2006). Physiological rehabilitation after video-assisted lung lobectomy for cancer: a prospective study of measuring daily exercise and oxygenation capacity. Eur J Cardiothorac Surg.

